# *Trichoderma* as a Model to Study Effector-Like Molecules

**DOI:** 10.3389/fmicb.2019.01030

**Published:** 2019-05-15

**Authors:** Claudia A. Ramírez-Valdespino, Sergio Casas-Flores, Vianey Olmedo-Monfil

**Affiliations:** ^1^División de Ciencias Naturales y Exactas, Departamento de Biología, Universidad de Guanajuato, Guanajuato, Mexico; ^2^Laboratorio de Biohidrometalurgia, Departamento de Medio Ambiente y Energía, Centro de Investigación en Materiales Avanzados, Chihuahua, Mexico; ^3^Laboratorio de Genómica Funcional y Comparativa, División de Biología Molecular, Instituto Potosino de Investigación Científica y Tecnológica, San Luis Potosí, Mexico

**Keywords:** *Trichoderma*, effector molecules, plant–microbe interactions, secondary metabolites, effector proteins, small RNA

## Abstract

Plants are capable of perceiving microorganisms by coordinating processes to establish different forms of plant–microbe relationships. Plant colonization is governed in fungal and bacterial systems by secreted effector molecules, suppressing plant defense responses and modulating plant physiology to promote either virulence or compatibility. Proteins, secondary metabolites, and small RNAs have been described as effector molecules that use different mechanisms to establish the interaction. Effector molecules have been studied in more detail due to their involvement in harmful interactions, leading to a negative impact on agriculture. Recently, research groups have started to study the effectors in symbiotic interactions. Interestingly, most symbiotic effectors are members of the same families present in phytopathogens. Nevertheless, the quantity and ratio of secreted effectors depends on the microorganism and the host, suggesting a complex mechanism of recognition between the plant and their associated microorganisms. Fungi belonging to *Trichoderma* genus interact with plants by inducing their defense system and promoting plant growth. Research suggests that some of these effects are associated with effector molecules that *Trichoderma* delivers during the association with the plant. In this review, we will focus on the main findings concerning the effector molecules reported in *Trichoderma* spp. and their role during the interaction with plants, mainly in the molecular dialogue that takes place between them.

## Introduction

One of the main challenges that agriculture production encounters today is to supply the demands of quality and quantity for the producer and consumer without affecting the environment. Various pathogenic agents attack the crops, among which filamentous fungi are the most destructive, causing important economic losses (Singh, 2014). To implement strategies to control plant diseases, it is necessary to understand the pathogenic process, determining how these fungi are established in plants and how they generate tissue damage and bypass plant defenses. In this sense, one of the main events currently under scrutiny corresponds to the early stages of the pathogen–plant interaction.

Now it is known that successful pathogens deliver a wide range of molecules to the plant, which allows them to overcome the obstacles presented by the plant, regarding perception, signaling, or active defense response (Di et al., 2016; Shen et al., 2018). Also, there is ambiguity about the classification of the molecules released by the pathogens, depending on the degree of response generated by the plant immune system and their impact on the pathogenic process (Thomma et al., 2011). Initially, the gene-for-gene model proposed that the interaction of an avirulent protein (Avr) from the pathogen with a plant counterpart, a resistant plant protein (R), triggers the plant defense response, avoiding disease progression. However, it has been determined that this interaction could also lead to the pathogenic processes, so it was redefined that avirulence factors can also be virulence factors (Flor, 1942; Bent and Mackey, 2007; Boller and Felix, 2009). For these pathogen-derived molecules, the term “effector” was proposed, defined as any given molecule that can alter the physiology, structure or function of another organism, facilitating the infection and/or triggering defense responses (Kamoun, 2006). This definition does not imply a positive or negative impact on the outcome of the host–pathogen interaction and therefore, can be applied to non-pathogenic interactions as well (Thomma et al., 2011).

In the plant–pathogen interaction, the participation of elicitors has also been reported, and those are described as a diverse group of molecules that induce plant defense in a weak and non-specific fashion and independent of races or cultivars (Alba et al., 2011). Additionally, it has been proposed that the participation of highly conserved pathogen-derived molecules, with essential functions in the pathogens, for example chitin and flagellin, are needed for the establishment of the interaction with the host. Overall, these molecules are termed Pathogen-Associated Molecular Patterns (PAMPs) and, since they have been described in non-pathogenic microorganisms, the more general concept of Microorganism-Associated Molecular Patterns (MAMPs) is also used (Jones and Dangl, 2006). Because of the wide distribution showed by elicitors, as well as their functional features, it was recently proposed to refer to them as PAMPs/MAMPs. In some cases, the distinction between PAMPs/MAMPs and effectors is not sufficiently defined, which is mainly related to the fact that PAMPs/MAMPs are highly conserved among genera, while effectors tend to be related to a single or few related species. However, new studies have uncovered that certain molecules classified as effectors are also widely distributed among several species (Thomma et al., 2011; Pazzagli et al., 2014). The debate about the functional classification of the molecules released by microorganisms remains open, and new evidence is necessary to clearly classify them and assign distinctive features for them. In order to avoid conceptual confusion, in this review, we will refer to effectors as those non-structural molecules derived from microorganisms that have a function modulating the plant defense pathways and/or participating in the establishment of associations with plants.

Plant defense response is based on the perception of PAMPs/MAMPs inducing primary PAMP- or MAMP-immunity (PTI/MTI), to counteract invading microorganisms (Jones and Dangl, 2006). The ‘effector-triggered immunity’ (ETI) is a second layer of defense, providing systemic resistance by sensing effectors from microorganisms to activate the induced systemic resistance (ISR), mediated by jasmonic acid (JA) and ethylene (ET). In this layer of defense, *PDF1.2* (Plant defensin 1.2), *Thi2.1* (Thionin) or *Chib* (Chitinase B) are commonly used as marker genes (Van Loon et al., 2006). Also, the systemic acquired resistance (SAR), regulated by salicylic acid (SA), leads to the expression of Pathogenesis-Related genes (PR) (Bari and Jones, 2009). Pathogens suppress the PTI producing a wide number of effectors with different functions, such as the prevention of the plant recognition or in the formation of infective structures (Kulkarni et al., 2005; de Jonge et al., 2010).

Traditionally, agriculture techniques to counteract phytopathogens involve the use of chemical formulations that have secondary effects such as toxicity and soil pollution. Safer agricultural strategies imply the use of biocontrol agents, displacing and eliminating phytopathogens. Among these agents, fungi classified in the *Trichoderma* genus are predominant. The antagonistic capacity of *Trichoderma* has been widely studied, and the mechanisms associated with it include competition for space and nutrients against its adversaries, antibiosis and mycoparasitism (Mukherjee et al., 2013).

Some *Trichoderma* species can effectively colonize plant roots and shoots and establish a molecular dialogue, having a positive effect in plants (Macías-Rodríguez et al., 2018; Manganiello et al., 2018). Mostafa and Gayed (1952) made the first observations in this regard, reporting that *Trichoderma* improves fresh and dry weight in cotton plants. More than 20 years later, Catska et al. (1975) reported that exudates from lettuce have a beneficial effect in conidia germination of *Trichoderma viride*, indicating that the fungus and plants obtain mutual benefits.

Moreover, an increasing amount of reports demonstrate that *Trichoderma* is an important plant endophyte that can interact with plants such as maize, cucumber, cotton, tomato, and *Arabidopsis thaliana* (Contreras-Cornejo et al., 2009; Vishnevetsky et al., 2010; Lopes et al., 2012; Mastouri et al., 2012; Reithner et al., 2014; Zhang et al., 2014; Lamdan et al., 2015). *Trichoderma* penetrates the first or second layers of cells of the epidermis in the root tissue or even colonizes intracellular spaces and grows between the plasma membrane and the plant cell wall (Yedidia et al., 1999; Nogueira-Lopez et al., 2018). Colonization by *Trichoderma* promotes plant growth, biomass gaining, higher seed germination, increased plant height, root development, shoot dry mass and leaf number, increased crop yield and improved plant vigor (Harman et al., 2004; Salas-Marina et al., 2015; Chagas et al., 2017). One of the most evident morphologic changes in plants triggered by *Trichoderma* is the increase of lateral roots, thus modifying root architecture. In this process, previous observations have demonstrated the participation of auxins (Contreras-Cornejo et al., 2009) as well as a cross-talk between ET and auxins through the signaling pathways mediated by MAP-kinases (Contreras-Cornejo et al., 2015). Also, the presence of *Trichoderma* not only modulates the levels of the hormones produced by the plant but *Trichoderma* itself can contribute with its own hormones or could provide intermediates for the synthesis of some phytohormones, as a part of the benefits reported in the *Trichoderma*–plant interaction (Guzmán-Guzmán et al., 2019).

Recent research has confirmed that these fungi activate plant defense pathways. However, how *Trichoderma* modulates the plant immune response to establish a beneficial interaction is one of the main challenges to be addressed. In the establishment of the beneficial association between *Trichoderma* and plants, the effectors may play key roles, as demonstrated in mycorrhizal systems such as *Laccaria bicolor* and *Glomus intraradices* (Kloppholz et al., 2011; Plett et al., 2011).

Here, we will focus on the filamentous fungi *Trichoderma* spp. and the efforts of the scientific community has done to identify their effector molecules, as well as their role in the establishment of a beneficial relationship with plants that promote growth and immune response activation.

## Plant Defense Response and Its Activation by *Trichoderma*

One of the first observations related with the *Trichoderma*-induced ISR in plants was the induction of the hypersensitive response (HR) and synthesis of phytoalexins in grapevine cell cultures after the application of a cellulase from *T. viride* (Calderón et al., 1994). *Trichoderma harzianum* also increases the resistance of *Phaseolus vulgaris* against *Botrytis cinerea* and *Colletotrichum lindemuthianum* (Bigirimana et al., 1997). *Arabidopsis* mutants, impaired in JA biosynthesis, showed a similar level of root colonization to wild-type plants (Martínez-Medina et al., 2017). The opposite effect was observed when impaired plants in the SA synthesis were analyzed. These plants were unable to restrict the colonization by the fungus, indicating that SA-mediated response is implicated in the regulation of the *Trichoderma*–plant colonization, thus preventing vascular system invasion (Alonso-Ramírez et al., 2014). These two results suggested that ISR does not play a relevant role during *Trichoderma* plant colonization, while SAR pathway modulates the root colonization extend.

Moreover, other studies indicate that ISR induced by *Trichoderma asperellum* in cucumber was associated with an increase in chitinase and peroxidase activity, as well as the modulation of genes that are implicated in the JA/ET signaling pathways (Yedidia et al., 2000; Yedidia et al., 2003; Shoresh et al., 2005).

*Trichoderma* can also induce SAR defense even when ISR defense is activated and they can improve the plant resistance against phytopathogens, such as *Sclerotinia sclerotiorum*, where the effect of *Trichoderma* strains correlates with the production of cell-wall degrading enzymes by the plant (Contreras-Cornejo et al., 2011; Salas-Marina et al., 2011; Lopes et al., 2012). In pepper, *Trichoderma stilbohypoxyli, Trichoderma Caribbaeum*, and *Trichoderma theobromicola* altered the expression of the genes involved in the hypersensitive response and sesquiterpene phytoalexins biosynthesis (Bae et al., 2010). In a tripartite system, involving pathogenic nematodes, *T. harzianum* T-79 and tomato plants, the presence of T-79 differentially primes the responses related to SA and JA in the plant. T-79 initially primes the SA pathway leading to a faster defense response, protecting the plant from the invasion. Later, the nematode suppresses the JA-mediated defense pathway, but T-79 initiates its priming activity on this pathway, antagonizing the pathogen and reducing its development and reproduction. Finally, when the infection by nematodes has already been established, T-79 intensely activates the SA pathway, increasing the defense against subsequent attacks by juvenile nematodes. These results show that the effects of T-79 over the plants are a dynamic phenomenon, which can be adapted to signals from other organisms in the near environment (Martínez-Medina et al., 2016).

## Proteins as Effectors in *Trichoderma*–Plant Interactions

Proteins were the first molecules proposed as effectors and have been widely studied in pathogenic systems. There is not much information about *Trichoderma* effector proteins, less than 20 effectors have been experimentally analyzed during the interaction with plant systems (Table 1), among them the following:

**Table 1:** Protein effectors from *Trichoderma* functionally validated during the interaction with plants.

Family	Protein	*Trichoderma* strain	Function in interaction with plants	References
Cerato-platanins	Sm1 (small protein 1)	*T. virens*	Induction of defense-related genes, production of ROS and phenolic compounds.	Djonovic et al., 2006, Djonovic et al., 2007; Salas-Marina et al., 2015
	Sm2 (small protein 2)	*T. virens*	Involved in root colonization and plant protection.	Crutcher et al., 2015
	Epl1 (eliciting plant response-like)	*T. atroviride*	Induction of defense-related genes.	Salas-Marina et al., 2015
		*T. harzianum*	Induction of defense-related genes.	Gomes et al., 2015
		*T. formosa*	Triggered of plant immune system.	Cheng et al., 2018
		*T. asperellum*	Induction of defense-related genes.	Yu et al., 2018
	Swollenin (expansin-like protein)	*T. asperellum*	Involved in root colonization and induction of plant defense.	Brotman et al., 2008
Glycoside-hydrolases	Thph1 and Thph2 (cellulase-like protein)	*T. harzianum*	Induction of defense-related genes.	Saravanakumar et al., 2016
	Cellulases	*T. longibrachiatum*	Activation of plant defense pathways.	Martínez et al., 2001
	ThPG1	*T. harzianum*	Involved on colonization of plant roots.	Morán-Diez et al., 2009
	Eix (xylanase)	*T. viride*	Triggers ET biosynthesis and hypersensitive response.	Rotblat et al., 2002
Hydrophobins	HYTLO1	*T. longibrachiatum*	Activate defense-related responses and growth promotion.	Ruocco et al., 2015; Moscatiello et al., 2018
	TVHYDII1	*T. virens*	Involved in colonization of plant roots.	Guzmán-Guzmán et al., 2017
	*HBF2-6*	*T. asperellum*	Involved in root colonization and induction of JA and SA pathways.	Huang et al., 2015
	*TasHyd1*	*T. asperellum*	Involved in root colonization.	Viterbo and Chet, 2010

*The effectors are divided by families, we indicate the strain from Trichoderma that produce the protein effector and their function in plants*.

### Cerato-Platanins

These are non-catalytic secreted proteins, which contribute to virulence in pathogens, acting as expansin-like proteins weakening cellulose aggregates from the cell wall (Baccelli et al., 2013). The Small Proteins (Sm) Sm1/2/3 from *Trichoderma virens* and their orthologs in *Trichoderma atroviride* Eliciting plant-response (Epl) Epl1/2/3 are produced in the association with plants, playing different roles. Transcripts and proteins corresponding to Sm1/Epl1 are detected as the most abundant in cultures supplemented with glucose or during the interaction with tomato or maize plants (Djonovic et al., 2006; Seidl et al., 2006; Gaderer et al., 2015). In maize, only the monomeric form of Sm1 or Epl1 proteins can induce the plant defense response and the dimeric form blocks the activation of ISR. When *T. virens* and *T. atroviride* strains were cultured in the presence of maize seedlings, Sm1 and Epl1 were produced as a glycosylated monomer and as a non-glycosylated dimer, respectively. These results suggest that the signals released by the plant influence the state of glycosylation and multimerization of Sm1/Epl1 proteins that may control the *Trichoderma–*plant molecular dialogue (Vargas et al., 2008). However, Sm2/Epl2 proteins are quantitatively fewer present that Sm1/Epl1 during the interaction with maize, there is evidence showing that Sm2 and Epl2 are more relevant in the activation of defense and root colonization in maize. The mechanism employed by Sm2/Epl2 to induce plant defense is unknown, but a strong reduction of the protection level of maize seedlings against *Cochliobolus heterostrophus* was observed when plants were treated with the *sm2*/*epl2* knockout strains in contrast with *sm1*/*epl1* knockout strains (Crutcher et al., 2015; Gaderer et al., 2015).

### Hydrophobins

These are small surface-active proteins, which are only found in fungi. The genomes from *T. virens* and *T. atroviride* contain 17 sequences encoding for hydrophobins (Kubicek et al., 2011), and the upregulation of some of them has been reported during *Trichoderma*–plant interactions. The expression of the hydrophobin-encoding gene *HFB2-6* was down-regulated in the presence of 1% poplar leaves powder, whilst the gene upregulated under 1% poplar root powder, suggesting that HFB2-6 has a function in root colonization. Moreover, the recombinant hydrophobin purified from *E. coli* activated the JA and SA signal transduction pathways when used on poplar seedling. Also, the upregulation of two genes related to auxin signaling was observed. Therefore, this protein seems to be involved in the promotion of growth and defense of poplar plants (Huang et al., 2015). TVHYDII1, from *T. virens*, participates in the colonization of tomato roots; this was demonstrated by using null and overexpressing *tvhydii1* strains (Guzmán-Guzmán et al., 2017). Likewise, the *TasHyd1* from *T. asperellum* participates in the colonization of cucumber plants (Viterbo and Chet, 2010). The purified hydrophobin HYTLO1 from *Trichoderma longibrachiatum* activates the defense response and promotes plant growth. Moreover, knockout strains significantly decreased their antagonistic activity and their capability to promote plant growth (Ruocco et al., 2015). Therefore, HYTLO1 has a dual role in the process of interaction with plants.

### Glycoside-Hydrolases

This is a wider group of proteins with enzymatic activity that are secreted by *Trichoderma*; some of them have been characterized and implicated in the *Trichoderma–*plant interaction. The xylanase Eix (ethylene induced xylanase) from *T. viride* triggers ET biosynthesis and the hypersensitive response in tobacco plants, highlighting that the elicitation of ET biosynthesis is not related with Eix enzymatic activity (Sharon et al., 1993; Rotblat et al., 2002). The cellulases, Thph1 and Thph2 from *T. harzianum*, were applied on maize leaves and this treatment led to the transient elevation of free cytosolic calcium and the production of reactive oxygen species (ROS). The Δ*Thph1*- or Δ*Thph2* null mutants were not able to upregulate the expression of genes related to the jasmonate/ET signaling pathway in maize. By using the yeast two-hybrid system, Thph1 and Thph2 can bind to the autophagocytosis associated protein (ZmATG3) and germin-like protein (ZmGL) of the plant, respectively. The identification of this molecular interaction opens the possibility to analyze the cellular localization of these targets as well as their participation in the ISR (Saravanakumar et al., 2016, 2018).

### CFEM and Small Secreted Cysteine-Rich Proteins (SSCPs)

In *Trichoderma* spp., one of the most abundant groups of secreted proteins corresponds to small proteins containing four or more cysteine residues. The SSCPs could be grouped into protein families with functions such as hydrophobins, some glycosyl hydrolases, cerato-platanins, CFEM proteins (Common in several Fungal Extracellular Membrane) as well as proteins with unknown function (Druzhinina et al., 2012).

Lamdan et al. (2015) reported a secretome analysis from the interaction between *T. virens* and maize, detecting 13 SSCPs negatively regulated by the presence of the roots. Plant inoculation with independent knockouts strains, for four of these genes encoding the SSCPs (ID 92810, ID 71692, ID 111486, and ID 77560), showed an improvement in ISR activity compared with the wild-type strain, without affecting root colonization. SSCPs could act as negative effectors reducing the defense levels in the plant and may be important for the fine-tuning of ISR by *Trichoderma*. Bioinformatic predictions indicated the abundant presence of SSCPs in *T. atroviride* and *T. virens* genomes, some of them containing CFEM domains, which are present in cell surface proteins with important roles in the interaction with other organisms (Pérez et al., 2011; Druzhinina et al., 2012). Possibly, but not in all cases, CFEM proteins participate as negative regulators of plant defense. In the bioinformatic analysis reported by Guzmán-Guzmán et al. (2017), there are 32 sequences grouped as CFEM; among them the *tacfem1* gene, which was upregulated when the fungus was co-cultivated with *A. thaliana*, suggesting a possible role during the establishment of the interaction. Also, in *T. virens*, the SSCP gene ID 19757 was upregulated in the interaction with both maize and tomato, while the ID 17705 gene was upregulated only by the presence of maize plants, rendering it a promising candidate for further analysis during the *T. virens*-maize interaction (Morán-Diez et al., 2015).

### Hunting for New *Trichoderma* Protein Effectors

One of the main strategies to identify effector proteins is to analyze their upregulation in the presence of plants (Djonovic et al., 2006; Plett et al., 2011; Guzmán-Guzmán et al., 2017). *Trichoderma* transcriptome and secretome analyses under different culture conditions led to describe that cellulases, small proteins, and cytochrome p450, among others, are highly represented (Lamdan et al., 2015; Morán-Diez et al., 2015; Rocha et al., 2016). Recently, a catalog of 233 putative effector proteins was reported from *T. virens, T. atroviride*, and *Trichoderma reesei*, identified and grouped in 18 families. The expression pattern of some of these genes was analyzed during the *Trichoderma–Arabidopsis* interaction, observing the upregulation of genes grouped in LysM proteins, Serine-proteases, Thioredoxins, Hydrophobins, CFEM, and Cerato-platanin families. Down-regulation was also observed, as in the case of the *Tvmp1* gene, encoding for a Metalloprotease (Guzmán-Guzmán et al., 2017), and for the *Tvcyt2* gene, which encodes a p450-cytochrome (Ramírez-Valdespino et al., 2018). However, more than 200 sequences from this *in silico* analysis were not studied further and their possible function during *Trichoderma*–plants interaction remains unknown.

The LysM effectors play relevant roles in the establishment of pathogenic interactions: they protect the fungal mycelium by either covering the surface of the hypha, thereby interfering with the enzymatic activity of the plant delivered chitinases or hijacking fungal cell wall derived chitin fragments, thereby avoiding the stimulation of the immune response (de Jonge et al., 2010; Kombrink et al., 2017). LysM encoding genes are present in *Trichoderma* genomes, but they are not characterized as well (Mendoza-Mendoza et al., 2018). In the catalog of putative effector proteins, there are 15 LysM encoding genes, six belonging to *T. atroviride* and nine to *T. virens*. Additionally, the *tvlysm1* gene was upregulated in co-culture of *T. virens* with *Arabidopsis*, suggesting a role during *Trichoderma* interaction with the plant (Guzmán-Guzmán et al., 2017). Additionally, the Tal6 protein from *T. atroviride*, was reported as an inhibitor of conidia germination (Seidl et al., 2013); however, their direct participation with plants has not been determined.

The differences in the experimental design in each case, where *Trichoderma*–Plant transcriptome/secretome are analyzed, makes it difficult to compare among all data sets to determine the functional relevance of these genes. In the case of *T. virens*, two secretomes obtained from hydroponic cultures, were analyzed looking for proteins differentially expressed during their interaction with maize seedlings. One of them analyzed the soluble proteins, while the other focused only on the proteins found in the plant apoplast. The main differences between both studies are related to the culture medium used and the time length of interaction. Lamdan et al. (2015) obtained the secretome from hydroponic cultures in sucrose-supplemented MS media after 96 h of interaction, a total of 280 soluble proteins were detected, of which 86% (241 proteins) contained a predicted signal peptide, identifying members of glycoside hydrolases (GH), LysM proteins, CFEM, lipases, and SSCPs, including Sm1 as the most abundant protein. From all secreted proteins, 66 were identified as differentially expressed, 32 were increased, while the remaining 34 showed a decrease when maize seedlings were present. Nogueira-Lopez et al. (2018) reported 43 secreted proteins, obtained from apoplastic space of maize roots cultured in Hoagland’s solution after 60 h of interaction with *T. virens*, and using a filtered-pipeline, nine of them were classified as putative effector proteins. Both secretomes share 13 putative candidates, being the GH group; the most representative proteins with five protein family members (Supplementary Table S1). The low number of common sequences could be due to the filtered-pipeline used in each study or due to the differences in growth conditions and sample collection times; without leaving aside the possibility that the roots could retain some proteins limiting their detection in the media (Lamdan et al., 2015).

The comparison between each secretome with the proposed *T. virens* effectors from the *in silico* analysis showed differences related to the number of effector candidates, which is expected given that the predicted secretome includes all sequences with the potential of being effectors.

Considering the full list of the apoplastic secretome (43 proteins), the proteins found with differential expression in the complete soluble secretome (66 proteins) and the effector candidates from the *in silico* prediction (84 proteins), we only found the CFEM member (ID 92810) and the GH member (ID 42143) in the three research works, while 22 common sequences were found between the soluble secretome (Lamdan et al., 2015) with the predicted effectors (Guzmán-Guzmán et al., 2017). Whereas, five sequences from the *in silico* analysis are present among the apoplastic secretome proteins, but they were not considered as effectors.

The differences observed among the experimental strategies to identify new protein effectors in the association of *Trichoderma* with plants suggests that there is still no consensus to consider products with the potential to participate as effectors, which represents a relevant topic to analyze further. The *in silico* analysis can be less precise when identifying effector molecules, but they provide a wide range of candidates to determine biological functions and the best way to know the function of a putative effector is to perform a wide and specialized characterization. Understanding and identifying the effector proteins will provide tools to deepen our understanding on how plant-beneficial fungi interaction is established and which could be the differences between a beneficial and a pathogenic system.

## Secondary Metabolites as Effectors in *Trichoderma*–Plant Interactions

One of the main characteristics of fungi is the production of a huge diversity of secondary metabolites (SMs); compounds with potential application in food, pharmaceutical and agricultural industries (Brakhage, 2013). It is generally assumed that SMs are not essential for growth and development in fungi but play important functions in the sensing, signaling and counteracting processes for the organisms present in their environment (Macheleidt et al., 2016). SMs comprise compounds of low molecular weight, diffusible in the culture medium or volatile, which are synthesized through a great variety of pathways (Brakhage, 2013). The synthesis of SMs is usually different between strains, and is influenced by growth conditions (Yu and Keller, 2005). At the molecular level, this environmental influence is related to the availability of regulatory elements. In *T. reesei*, the SOR cluster contains two genes encoding for transcription factors involved in biosynthesis of sorbecillinoids, a group of yellow pigments with antimicrobial activities. YPR2, one of those transcription factors, carries out its major function in constant darkness and in the presence of cellulose as a carbon source. The function of YPR is positively related to the levels of alamethicin and to the production of orsellinic acid in darkness (Hitzenhammer et al., 2019). In *T. atroviride* and *T. virens*, it has been shown that heterotrimeric G proteins, mitogen-activated protein kinases (MAPK) and transcription factors are involved in the signaling pathway leading to SMs synthesis, which also respond to environmental conditions as a type of nutrients, pH, light or temperature (Reithner et al., 2005; Mukherjee and Kenerley, 2010). Antibiotic activity has been reported for SMs produced by *Trichoderma* species against various yeast, filamentous fungi and bacteria, causing growth inhibition or cell death. These SMs, acting synergistically with hydrolytic enzymes, are more likely to be implicated in the effectiveness of the strain producing them as a biological control agent (Reino et al., 2008).

Although the metabolites produced by plants could also affect the association with microorganisms, either by favoring it or by restricting it, in this section we will focus on those SMs produced by *Trichoderma* species that have shown impact in their capacity as plant symbionts.

### Lactones

These are generally very pleasant, potent, flavor materials, which are widely distributed in nature (Kapfer et al., 1989). The best-known SM for *Trichoderma* species is 6-pentyl-2H-pyran-2-one (6-PP), derived from linoleic acid and the pathway of its formation has been elucidated by metabolization of [U-14C] and of [1-14C] linoleic acid in *T. harzianum* (Serrano-Carreon et al., 1993). In plants, 6-PP interferes with the signaling pathway involving auxins and ET, promotes plant growth, and regulates root architecture by inhibiting the growth of the primary root and inducing the formation of lateral shoots, by modulating the expression of genes encoding for auxin transporters. The modification related to the lateral shoots is mediated by the TIR1, AFB2, and AFB3 receptors and the ARF7 and ARF19 transcription factors, while the sensitization in the main root is mediated by EIN2 (Garnica-Vergara et al., 2016). In field, the application of 6PP (1 μM) or a spore suspension of *T. harzianum* T22 (10^8^ spore/liter) in *Vitis vinifera*, increased polyphenol content, antioxidant activity and weight in fruits (Pascale et al., 2017). Cremenolide is a 10-member lactone isolated from *Trichoderma cremeum*, that improved the root length and fresh weight in tomato seedlings, but changes in plant height were not observed (Vinale et al., 2016).

### Peptaibols

These are small linear peptides of non-ribosomal synthesis, which usually have a high content of 2-amino-isobutyric acid (Aib) bound to unconventional amino acids, such as ethyl-valine, isovaline, and hydroxyproline. There are at least 190 compounds of this type synthesized by *Trichoderma* species^1^.

In *T. virens*, 18-mer peptaibols are produced through the activity of the Non-Ribosomal Peptide Synthase (NRPS) encoded by the gene *Tex1*. This kind of peptaibols activates the plant defense in *Cucumis sativus* against *Pseudomonas syringae*. Cucumber plants growing in contact with *T. virens* increase the expression of three genes involved in the synthesis of phytoalexins, *hpl, pal1*, and *prx*, which encode for a hydroxy peroxide lyase, phenylalanine ammonia lyase, and peroxidase, respectively. The *tex1* null mutants lose their ability to produce 18-mer peptaibols, leading to a lower expression of *hpl, pal1*, and *prx* genes in plants. The use of two synthetic peptaibols TvBI and TvBII on cucumber seedlings activated systemic protection against bacteria and induced the expression of *hpl, pal1*, and *prx* (Viterbo et al., 2007). Null mutants in *ppt1* gene, which codes for a 4-phosphopantetheinyl transferase in *T. virens*, are affected in the synthesis of 11, 14, and 18-mer peptaibols, although their capability to colonize roots is not affected. Furthermore, antibiosis on phytopathogens, as well as the ability to induce the synthesis of SA and of camalexin in *Arabidopsis* plants, is compromised in these strains (Velázquez-Robledo et al., 2011).

Alamethicin is one of the most studied peptaibols due to its ability to induce defense responses in plants such as callose deposition, expression of genes related to plant defense, production of SMs, and accumulation of phenolic compounds, that together can improve the vigor of plants and their response to stressing conditions (Rippa et al., 2010). In *A. thaliana*, the presence of alamethicin interferes with the synthesis of methy farnesoate (MeFA), an SM related to herbivory, by altering the presence of the *miRNA163*, which targets genes encoding methyltransferases relevant in the synthetic pathway of the MeFA (Ng et al., 2011). It is proposed that alamethicin increases ion permeability in the cell membrane (Duclohier and Wróblewski, 2001). Trichokonin VI (TK VI) is produced by *T. longibrachiatum* SMF2, and it has been shown that this peptaibol interferes with the GORK channel, a rectifying K+ channel gated outwardly, which alters the root structure by inhibiting cell division and elongation in the main root. TK VI increases the auxin content and interrupts its gradient at the tips of the roots, interfering with the local synthesis and its polar transport (Shi et al., 2016).

### Polyketides

These are one of the most abundant groups of SMs in nature, which includes macrolides, polyenes, and polyphenols. They have been studied in detail because the group includes compounds with an impact on human health such as sterigmatocystin, aflatoxin B1 and lovastatin. They are produced by polyketide synthases (PKSs), multi-domain proteins similar to fatty acid synthases, which condense acetyl coenzyme A or malonyl coenzyme A units, to form carbon chains of variable length (Chiang et al., 2010). In *Trichoderma arundinaceum*, the production of 4 aspinolides has been reported, in particular, aspinolide C participates in the induction of genes *PR1b1, PR-P2* involved in the signaling pathway mediated by SA. During the interaction of *T. virens* with maize plants, the expression of the defense-related genes *pal1* and *aos* (allene oxide synthase) is increased. This upregulation is related to SMs produced by *T. virens* through the activity of the PKS/NPRS encoded by the *Tex 13* gene. Strains affected in *Tex13* retained their ability to increase the expression of the gene *aos* in plant, but they could not upregulate the *pal1* gene (Mukherjee et al., 2012).

### Terpenes

These are a highly diverse family of SMs at the structural and stereochemical level. These molecules are derived from long polyisoprenoid diphosphates that can be cyclized to generate single or multiple ring products. The cyclization reactions are carried out by high-affinity terpene cyclase enzymes, which generate a single product or by promiscuous enzymes that can generate up to 52 different products (Christianson, 2008). Cytochrome p450 enzymes are involved in the reactions of synthesis and/or modification of terpenes. Recently 477 cytochrome P450s have been identified from seven *Trichoderma* species (Chadha et al., 2018). Cytochrome p450 activity is needed for the synthesis of SMs, which are related to the mycoparasitic capacity and/or its association with plants. The enzyme encoded by the *G3* gene in *Trichoderma hamatum* is activated in response to *Sclerotinia* and *Sclerotium* species (Carpenter et al., 2008). Through the generation of mutant and overexpressing strains of the *Tvcyt2* gene of *T. virens*, five terpene-like compounds were identified as involved in the antagonistic activity against *Rhizoctonia solani*, in the activation of genes related to the JA pathway in *Arabidopsis* plants as well as in the promotion of growth in *Arabidopsis* and tomato seedlings (Ramírez-Valdespino et al., 2018).

### Trichothecenes

*Trichoderma brevicompactum* produces trichodermin through a pathway involving the activity of p450 enzyme, encoded by *Tri5* gene. The overexpressing strains of this gene had a negative effect in tomato plants, decreasing the root length and plant size (Malmierca et al., 2015). Harzianum A (HA), isolated from *T. arundinaceum*, did not show any effect over growth of tomato seedlings. However, mutant strains that do not produce HA were impaired to upregulate the expression of genes involved in plant defense at the same level. HA could be sensitizing the plant cell to induce those genes faster and at higher levels (Malmierca et al., 2012).

### Volatile Organic Compounds (VOCs)

The VOCs group includes several small compounds with different chemical natures, playing relevant roles as essential signals in interactions among plant roots, microbes, and insects (Schenkel et al., 2015). The effect by the VOCs produced by 25 different *Trichoderma* strains on the plants were analyzed using *A. thaliana* as a host, in two independent research works. The general results showed that one of the strains produced VOCs with a negative impact on plant growth, 10 of the strains did not have any obvious effect on the plants, while fourteen of them had a positive effect on total biomass and on chlorophyll content. The analysis of the VOCs produced by each strain determined the presence of great diversity of compounds, suggesting the participation of several mechanisms to generate the final effect on plants (Lee et al., 2016; Nieto-Jacobo et al., 2017). This blend of compounds present in VOCs makes it difficult to determine which of the metabolites is responsible for the effects observed on the plants. Some strategies that could help to define their biological role are based on genetic manipulation in order to generate strains affected in key elements of the VOCs biosynthesis. Trichodiene (TD) is a VOC used as a substrate by the sesquiterpene synthase Tri5 to produce the compound Harzianum A (HA) in *T. arundinaceum*. The heterologous expression of the gene *Tri5* in *T. harzianum* led to the production of TD. VOCs released by this *Tri5*-transformant and TD itself induced the expression of tomato defense genes related to SA (Malmierca et al., 2015). In *T. atroviride*, the mutation of genes encoding membrane-bound NADPH oxidases (Nox), leads to the alteration of VOCs profiles. Loss of function of Nox1 or the regulator NoxR, in the presence of a functional Nox2 enzyme leads to the production of VOCs with inhibitory effects on plant growth (Cruz-Magalhães et al., 2019).

### Phytohormones

Phytohormones are important growth regulators with relevant roles on metabolism and plant defense responses. Several root-associated microbes are able to produce phytohormones that have an effect in plants (Egamberdieva et al., 2017). *T. virens* produces Indole-3-acetic acid (IAA) and indole-3-acetaldehyde (IAAId), which promotes plant growth and development in *A. thaliana* (Contreras-Cornejo et al., 2009).

Cytokinins (CKs) are essential molecules that regulate plant growth and development (Osugi and Sakakibara, 2015). ET is a volatile hormone that regulates a range of processes, from seed germination, organ senescence, among others (Bleecker and Kende, 2000). CKs promote hyphal branching and help in oxidative stress tolerance in *Magnaporthe oryzae* (Chanclud et al., 2016), whilst in mycorrhizal fungi, ET affects spore germination and growth (Barker and Tagu, 2000). There are no reports in *Trichoderma* about CKs or ET production, though in the genome of *T. atroviride*, the genes necessary to synthesize both compounds are present, suggesting that it could produce these phytohormones, with a potential impact in the association with plants (Guzmán-Guzmán et al., 2019). Abscisic acid (ABA) is involved in seed dormancy and development, abiotic stress response, among other roles in plants, and there is evidence of its production by several fungi on which ABA was proposed as a factor promoting plant colonization (Charpenter et al., 2014; Chanclud and Morel, 2016). There were six genes related to the ABA biosynthesis pathway identified in *T. atroviride*. However, no homolog to *bcaba3/ataba3*, a gene encoding a key enzyme in last steps of the biosynthesis of ABA was found, suggesting that *T. virens* is unlikely to produce this phytohormone and that it could only provide an ABA intermediary (Guzmán-Guzmán et al., 2019). Additionally, it was proposed that *T. virens* and *T. atroviride* modulate ABA-regulated responses, such as stomatal aperture and leaf transpiration in *A. thaliana* (Contreras-Cornejo et al., 2015).

### Factors That Influence the Effect of SMs on Plants

Three relevant factors related to the positive effect generated on the plants by SMs are, the dose used of each SM, the physiological state, as well as the genetic background of the plants tested (Vinale et al., 2009; Li et al., 2019). The effect of alamethicin over plants has been tested at high doses such as 10 and 50 mM. When these are applied to *Arabidopsis* seedlings, they generate plant death and this toxicity is related to cleavage of ribosomal RNA and cellular lysis. The use of alamethicin at lower doses (5 μM) suggest that a threshold in the concentration of this SM is required to trigger only plant-cell permeabilization and induce programed cell death as a hypersensitive response, showing similarities with the response elicited by avirulent pathogens or by compounds that mimic a pathogen attack (Rippa et al., 2007; Rippa et al., 2010). Koninginins A, B, C, E, and G tested at 10^-3^ M, inhibited growth of etiolated wheat coleoptiles at different rates, varying between 54 and 65% and up to 100% (Reino et al., 2008). Treatments of 6-PP over *A. thaliana* seedlings in doses from 50 to 175 μM increased shoot, root and total plant biomass while at 200 μM, no increase in biomass was observed. 6-PP also raised both lateral root number and density in a dose- dependent manner, showing an inhibition of primary root growth from 125 μM onwards, without cell damage (Garnica-Vergara et al., 2016).

The plant genetic background is a determinant in the response generated by specific strains of *Trichoderma*. In maize, the use of *T. harzianum* T22, one of the most widely used commercial strains, induced strong positive growth over eight maize hybrids tested, it had little effect on growth over eight other hybrids and it even negatively affected growth of two other maize hybrids (Harman, 2006). While in tomato, independent symbiotic interactions between *T. harzianum* T22 or *T. atroviride* P1 with four *Solanum lycopersicum* lines or the wild *Solanum habrochaites* accession demonstrated that genetic variability is a determinant in the response shown by plants related to growth, weight, resistance against *B. cinerea* and the expression of genes involved in plant defense (Tucci et al., 2011).

We propose that many SMs can be classified as effectors and the different *Trichoderma*–plant systems developed by different work groups make evident the utility of using them as a biological model. Metabolomic analysis, involving the study of the concentrations, structures and interactions of thousands of SMs represents a useful tool to identify molecules with potential biotechnological application in the improvement of plant yield and vigor.

## *Trichoderma*’s Small RNAs as Putative Effectors

Thus far, plant analysis has focused on the identification of proteins involved in the plant immune response. However, several lines of evidence have shown that plants also use non-coding RNAs against pathogens (Pumplin and Voinnet, 2013) and symbionts (Pieterse et al., 2014). Small RNAs (sRNAs) play important roles in plant immune responses against virus, bacteria, fungi, and oomycetes (Pumplin and Voinnet, 2013). sRNAs are 20–30 nucleotide long non-coding, sequence specific regulatory RNA molecules that mediate gene silencing to regulate physiological and developmental processes (Chang et al., 2012; Pumplin and Voinnet, 2013). In plants, sRNAs are processed from double-stranded or single-stranded RNA with hairpin structures by Dicer-like (DCL) proteins, which release RNA duplexes. After processing, sRNAs are loaded into RNA-induced silencing complexes (RISC), which contains one member of the ARGONAUTE (AGO) protein family, leading to transcriptional gene silencing by guiding heterochromatin formation, inhibiting mRNA translation or inducing mRNA degradation. Several members of the sRNA biogenesis machinery are involved in plant immunity, including DCL, AGO, DCL associated proteins and RNA dependent RNA polymerases (RDRs). Mutants in such genes show defects in sRNAs accumulation and are impaired in pathogen response (Seo et al., 2013).

During the last decade, cross-kingdom RNA interference (RNAi) between host and phytopathogens has demonstrated its role in the successful colonization of plant tissues by the phytopathogens or in their avoidance by the plant. For instance, host-induced gene silencing (HIGS), a technology developed to protect crops from fungal infections by expressing dsRNA *in planta* to silence virulence genes, enhances plant resistance to *Fusarium verticillioides* or *Blumeria* spp. upon infection of the host plant (Nowara et al., 2010; Tinoco et al., 2010). HIGS technology is also effective against *Puccinia* spp. (Yin et al., 2011, 2014), *Fusarium* spp. (Koch et al., 2013; Hu et al., 2015) and *S. sclerotiorum* (Andrade et al., 2016).

*B. cinerea*, produces complementary sRNAs against plant immunity related genes in *Arabidopsis* and *Lycopersicum esculentum* (Weiberg et al., 2013). Upon infection of *Arabidopsi*s by *B. cinerea*, the fungal sRNAs associate with *Arabidopsis* AGO1 protein, interfering with plant target mRNAs, for example, those encoding MAPKs. These findings indicate that the fungus hijacks the host RNAi machinery to silence the host own genes (Weiberg et al., 2013; Wang M. et al., 2017). Moreover, *Arabidopsis ago1-27* mutants are more resistant to *B. cinerea* infection, supporting that the fungus uses the plant RNAi machinery to silence host genes. Transferred fungal sRNAs into the plant cell are detected in *B. cinerea dcl1* or *dcl2* single mutants, but not in a *dcl1/dcl2* double mutant, leading to a diminished fungal virulence, indicating that the biogenesis of sRNAs is required for pathogenesis (Weiberg et al., 2013). Transgenic plants expressing one of these sRNAs (Bc-siR37) silence *Arabidopsis* genes encoding a pectin lyase, a WRKY transcription factor, and a receptor-like kinase (Wang M. et al., 2017). The causal agent of wheat stripe rust disease, *Puccinia striiformis*, silences the mRNA that encodes for the PR-2 protein, through Pst-milR1, a miRNA-like sRNA (Wang B. et al., 2017). In *Verticillium dahliae*, it was shown that AGO2 does not play a role in the infection of *Arabidopsis.* This pathogen infected *Arabidopsis ago2*-1 normally, whereas in *ago1*-*27* mutant, the rate of infection was reduced (Wang et al., 2016).

Successful establishment of the *Trichoderma*–plant interaction during early stages of root colonization implies the activation of cell detoxification and protection mechanisms in the fungi (Ruocco et al., 2009; Estrada-Rivera et al., 2019). Therefore, these fungi possess effective systems that efficiently scavenge harmful compounds from the cell. This has been partially shown with the suppression of phytoalexin and plant genes related to the defense mechanisms in *Lotus japonicus* and *Arabidopsis* during its interaction with *Trichoderma koningii* and *T. atroviride*. Application of these fungi to plant roots induced a rapid accumulation of host transcripts that encode key enzymes of SAR and ISR and those involved in phytoalexin synthesis. The expression of these genes is transient and decreased to levels of the control plants. *Trichoderma* resembles mycorrhizal fungi in the establishment of symbiotic associations rather than fungal pathogens (Masunaka et al., 2011; Estrada-Rivera et al., 2019). Production of sRNAs by filamentous fungi, including *Trichoderma* species, has been documented. Although analysis of mutants in the biogenesis components of sRNAs in fungi have shown their role on their biology (Chang et al., 2012; Carreras-Villaseñor et al., 2013), much is yet to be explored regarding the roles of sRNAs in this kingdom. There is no direct evidence for the role of beneficial microorganisms sRNAs on the suppression of plant immunity to establish a symbiotic relationship. In our group, we sequenced sRNAs libraries during *Arabidopsis*–*T. atroviride* interaction at different interaction times. Mapping the sRNAs over the *T. atroviride* genome revealed that 37 sRNAs of the fungus matched with genes of the host plant. Interestingly, target genes encode lytic enzymes, MAPKs putatively involved in plant immunity, proteins with a domain of unknown function, disease resistance protein, NBS-LRR class family proteins, and *S*-Adenosyl-L-Methionine-dependent methyltransferases superfamily proteins, among others. This indicates that *T. atroviride* could be using sRNAs as effector molecules, similarly to *B. cinerea* and *V. dahliae*, to establish a symbiotic relationship with *Arabidopsis* through interfering mRNAs involved in plant immunity, chromatin modifications and cell wall degrading enzymes, among others that remain to be determined. However, more research is needed to unravel the molecular mechanisms mediated by the fungal sRNAs that allows us to better understand the mechanisms that lead to the establishment of this beneficial association.

## Conclusion

**FIGURE 1:**
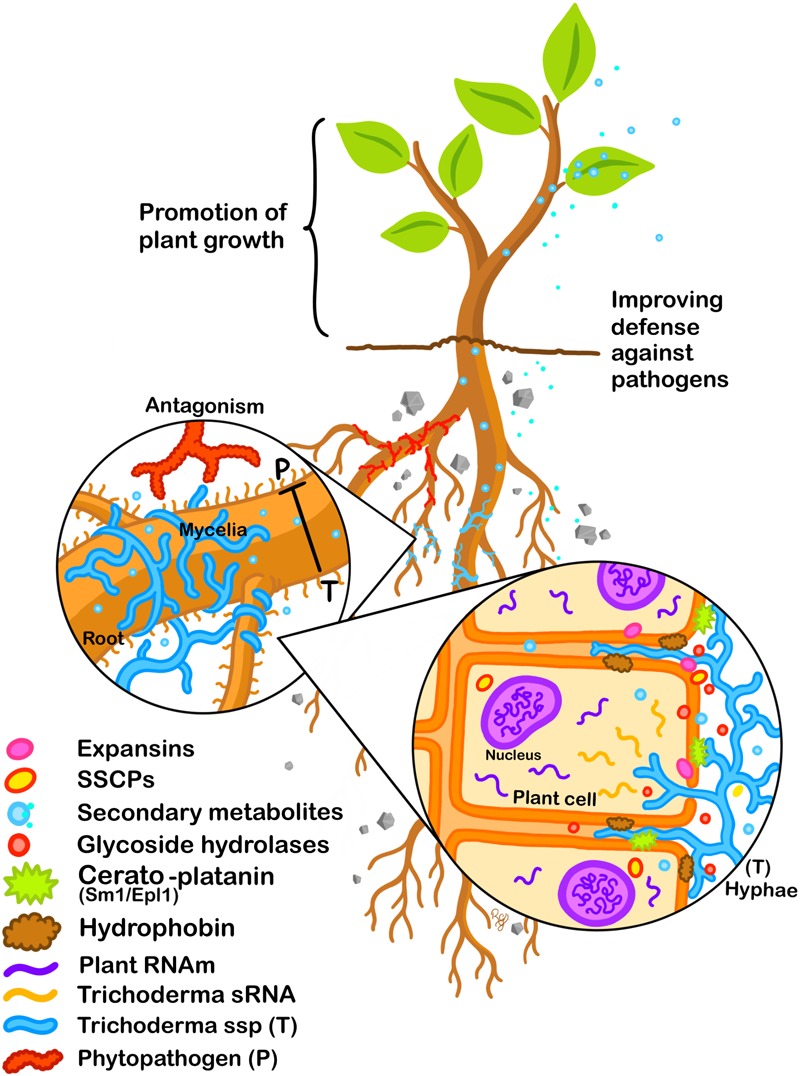
Effectors participate during *Trichoderma*–plant interaction. Antagonist activity of *Trichoderma* against phytopathogenic fungi. The release of molecules from *Trichoderma* with activity as effectors is highlighted. These molecules will modulate the plant hormonal balance as well as its defense response, allowing colonization. The beneficial association will result in the improvement of plant growth and in the resistance against phytopathogens.

The consequence in the field of the presence of *Trichoderma* on plants can be indirect, for example by exerting antagonistic activity on potential phytopathogens, by attacking them, and by colonizing the rhizosphere, so they can avoid the contact of the pathogen with plant tissue. Direct beneficial effects on plants by *Trichoderma* are related to root colonization, although in many cases it has been shown that direct contact may not be necessary. Effectors of *Trichoderma* may play a key role in the success of colonization of the plant, first by establishing the initial contact and subsequently maintaining the fungus–plant interaction (Figure 1). Many of the effectors that have been identified in *Trichoderma*, and in other fungal symbionts, have reported activities described in their pathogenic counterparts. Therefore, the success of the interaction must not only rely on molecules playing these functions. The physiological state of both participants, as well as their threshold of perception toward the molecular signals exchanged are also important factors. This regulation does not depend solely on the genetic background of the fungus, the signals generated by the plant are also important. The study of effectors in *Trichoderma* is a relatively recent topic; however, thanks to technological advances to detect, identify and quantify molecules (proteins, secondary metabolites, and RNAs), the scientific community already has an extensive catalog of effector candidates. In most cases, it is necessary to carry out biological validation and determine the spectrum of action of these effectors on different plants at the level of cultivars, species and even genera. Moreover, the analysis of the interaction, considering longer times, may indicate how *Trichoderma* can change the plant physiology, during its complete life cycle. Due to the versatility shown by *Trichoderma* to associate with a wide variety of plants, it would be possible to determine the relevance of those candidates in different plant systems. In this review, we have focused mainly on the participation of the effectors in the interaction with plants, but its relevance in fungus–fungus associations is still pending, highlighting the idea of a multidirectional molecular exchange in the rhizosphere.

## Author Contributions

CR-V, SC-F, and VO-M contributed equally with ideas and discussion material. VO-M coordinated the work. CR-V wrote the review.

## Conflict of Interest Statement

The authors declare that the research was conducted in the absence of any commercial or financial relationships that could be construed as a potential conflict of interest.

## Footnotes

1 http://peptaibol.cryst.bbk.ac.uk/home.shtml
