# Supplementary material for: Trichoderma as a Model to Study Effector-Like Molecules
Source: Front Microbiol. 2019 May 15;10:1030. doi: 10.3389/fmicb.2019.01030 (PMC6529561; doi:10.3389/fmicb.2019.01030)
Supplement: Supplementary file 1 [file Table_1.DOCX]

**Supplementary data:**

**Table I: *Trichoderma virens* proteins involved in plant interaction shared between secretomes and *in silico* analysis.** Nomenclature used by Guzmán-Guzmán *et al.,* (2017) was taken to name each of the families, excluding the SSP (ID 93159) which name was taken from Lambdan *et al.,* (2015). Green, orange and gray shadows indicate up-regulation, down-regulation and no differential products, respectively.

| **Family** | **JGI ID** | **Guzmán *et al., 2017*** | **Nogueira *et al.,* 2018** | **Lambdan *et al., 2015*** |
| --- | --- | --- | --- | --- |
| LysM | 201746 | Yes |  | Yes |
| Thioredoxins | 111061 | Yes | Yes |  |
| CFEM domain | 82827 | Yes |  | Yes |
|  | 92810 | Yes | Yes | Yes |
| Cerato-platanins | 110852 | Yes | Yes | Yes |
| Serine-proteases | 77334 | Yes |  | Yes |
|  | 41430 | Yes |  | Yes |
|  | 88460 | Yes |  | Yes |
|  | 69939 | Yes |  | Yes |
|  | 87425 | Yes |  | Yes |
|  | 180959 | Yes |  | Yes |
|  | 71933 | Yes |  | Yes |
|  | 90003 | Yes |  | Yes |
| Metalloproteases | 86763 | Yes |  | Yes |
| Glycoside hydrolases | 42143 | Yes | Yes | Yes |
|  | 110754 |  | Yes | Yes |
|  | 29366 |  | Yes | Yes |
|  | 71600 |  | Yes | Yes |
|  | 90504 |  | Yes | Yes |
| Aspartil - proteases | 15627 | Yes |  | Yes |
|  | 34671 | Yes |  | Yes |
|  | 36107 | Yes |  | Yes |
|  | 81434 | Yes |  | Yes |
|  | 196192 | Yes |  | Yes |
|  | 213726 | Yes |  | Yes |
| Cupin_1 | 50666 | Yes | Yes |  |
| Prokar lipoproteins | 77560 | Yes |  | Yes |
| Galactose mutarose-like protein | 87809 |  | Yes | Yes |
| Glutathione reductase | 54541 |  | Yes | Yes |
| L-domain-like protein (Ecm33) | 72615 |  | Yes | Yes |
| S-adenosylhomocystein hydrolase | 82877 |  | Yes | Yes |
| Superoxide dismutase [Cu-Zn] | 183329 |  | Yes | Yes |
| Oxidoreductases | 138628 |  | Yes | Yes |
| SSP | 93159 | Yes |  | Yes |
